# Molecular Identification and Geometric Morphometric Analysis of *Haematobosca* *aberrans* (Diptera: Muscidae)

**DOI:** 10.3390/insects11070451

**Published:** 2020-07-16

**Authors:** Tanasak Changbunjong, Jiraporn Ruangsittichai, Gerard Duvallet, Adrian C. Pont

**Affiliations:** 1 Department of Pre-clinic and Applied Animal Science, Faculty of Veterinary Science, Mahidol University, Nakhon Pathom 73170, Thailand; 2 The Monitoring and Surveillance Center for Zoonotic Diseases in Wildlife and Exotic Animals (MoZWE), Faculty of Veterinary Science, Mahidol University, Nakhon Pathom 73170, Thailand; 3 Department of Medical Entomology, Faculty of Tropical Medicine, Mahidol University, Bangkok 10400, Thailand; jiraporn.rua@mahidol.ac.th; 4 UMR5175 CEFE, Centre d’Ecologie Fonctionnelle et Evolutive, Université Paul-Valéry, 34090 Montpellier, France; gerard.duvallet@univ-montp3.fr; 5 Oxford University Museum of Natural History, Parks Road, Oxford OX1 3PW, UK; muscidman2@gmail.com

**Keywords:** cytochrome c oxidase I, geometric morphometrics, *Haematobosca*, sexual dimorphism, Stomoxyinae, Thailand

## Abstract

The genus *Haematobosca* Bezzi, 1907 (Diptera: Muscidae) contains haematophagous flies of veterinary importance. A new fly species of this genus was recognised from northern Thailand based on morphological characters and described as *Haematobosca aberrans* Pont, Duvallet & Changbunjong, 2020. In the present study, the mitochondrial cytochrome c oxidase I (COI) gene was used to confirm the morphological identification of *H. aberrans*. In addition, landmark-based geometric morphometrics was used to determine sexual dimorphism. The molecular analysis was conducted with 10 COI sequences. The results showed that all sequences were 100% identical. The sequence was not highly similar to reference sequences from GenBank and did not match any identified species from Barcode of Life Data Systems (BOLD). Phylogenetic analysis clearly differentiated this species from other species within the subfamily Stomoxyinae. For geometric morphometric analysis, a total of 16 wing pictures were analysed using the landmark-based approach. The results showed significant differences in wing shape between males and females, with a cross-validated classification score of 100%. The allometric analysis showed that wing shape has no correlation with size. Therefore, the COI gene is effective in species identification of *H. aberrans*, and geometric morphometrics is also effective in determining sexual dimorphism.

## 1. Introduction

*Haematobosca* Bezzi, 1907 (Diptera: Muscidae) is a genus of haematophagous flies in the subfamily Stomoxyinae. The genus comprises 15 species: 11 species in the Afrotropical region, one in the Palaearctic region, two in the Holarctic region and one in the Oriental and Australasian regions [1,2,3,4]. All the species in this genus are of considerable veterinary importance, affecting both domestic animals and wildlife [1,5]. The adult flies are medium-sized flies, 3.5 to 9.0 mm in length and yellow to black in colour. The arista has dorsal and ventral hairs. The palpi are about as long as the proboscis and are grooved internally. Anterior and posterior katepisternal setae (or sternopleural bristles) are present [1]. Recently, a new species of *Haematobosca* was described from Thailand as *Haematobosca aberrans* Pont, Duvallet & Changbunjong, 2020 [6]. It can be distinguished from other *Haematobosca* by the absence of the anterior katepisternal seta [6]. However, one female that probably belongs to this species was previously recorded incorrectly as *Stygeromyia* Austen, 1907 by Tumrasvin and Shinonaga (1978) [7].

The DNA-based identification system is a useful tool for species identification, biodiversity monitoring and molecular phylogenetic investigation [8,9]. The mitochondrial cytochrome c oxidase subunit I (COI) gene with a length of about 650 base pairs (bps) is commonly used as the DNA barcode of animal species. The COI gene has been found to be effective for the identification of various insects of medical and veterinary importance such as black flies [10,11], mosquitoes [12,13,14,15], sand flies [16,17,18,19] and tabanids [20,21,22,23]. The efficiency of the COI gene for the identification of stomoxyine flies has been demonstrated by Changbunjong et al. (2016) [24].

In parallel to DNA-based identification, geometric morphometrics is also increasingly applied to distinguish morphologically similar species of insects of medical and veterinary importance [25,26,27,28,29,30]. This technique is a fast, low-cost and easy to use tool [25]. Geometric morphometric analysis can be conducted using various methods such as landmark and outline-based methods, depending on the characteristics and specifics of the specimens [25,31]. The landmark-based method uses the coordinates of landmarks to analyse the variation in size and shape, while the outline-based method uses the outline or contour data in the analysis [25,31]. Many studies have used geometric morphometric methods for describing sexual dimorphism in the wing of various insects such as blow flies [28,32], mosquitoes [33,34], moths [35,36], fruit flies [37] and stomoxyine flies [26]. Sexual dimorphism in morphological characters is the most interesting source of phenotypic variation in various taxa and has attracted considerable interest in evolutionary biology [37]. Therefore, in our study, the COI gene was used as the molecular marker for the identification of *H. aberrans*, collected in northern Thailand. In addition, geometric morphometric analysis was used to determine sexual dimorphism.

## 2. Materials and Methods

### 2.1. Ethics Statement

Our protocol for specimen collection was approved by the Faculty of Veterinary Science, Mahidol University Animal Care and Use Committee (Ref. MUVS-2018-10-58).

### 2.2. Specimen Collection and Identification

**Figure 1:**
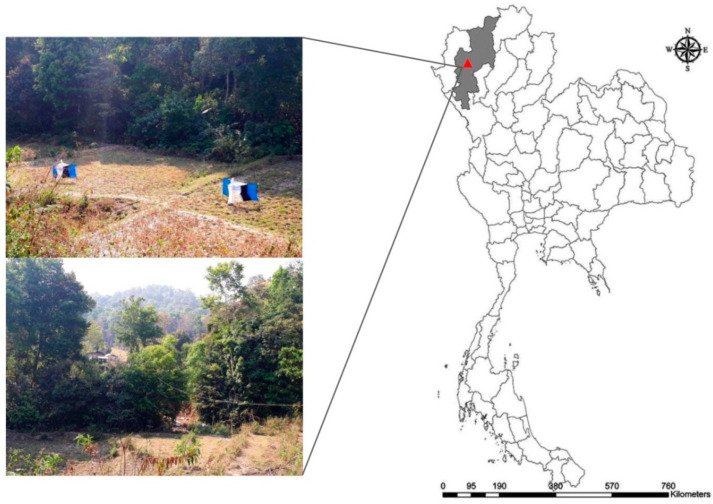
Map showing the collection site of *Haematobosca aberrans* Pont, Duvallet & Changbunjong, 2020 in Thailand.

Specimens of *Haematobosca aberrans* were collected in the Mae Wang district, Chiang Mai Province of northern Thailand (18°38′35″ N, 98°31′27″ E) in January 2019 using Nzi traps [38] (Figure 1). All flies were collected inside a buffalo farm close to a forest area. They were euthanised in a freezer (−10 °C) and placed in individual 1.5 mL microcentrifuge tubes. They were identified using the description and taxonomic key in Pont et al. (2020) [6]. Specimens were stored at −20 °C for further laboratory investigation.

### 2.3. Molecular Analysis

#### 2.3.1. DNA Extraction and COI Amplification

Genomic DNA was extracted from the legs of individual specimens using DNeasy^®^ Blood & Tissue Kit (QIAGEN, Hilden, Germany) according to the manufacturer’s protocol. PCR amplification of the COI gene with a 658 base pair (bp) fragment was performed using a primer pair consisting of LepF1 (5′-ATTCAACCAATCATAAAGATATTGG-3′) and LepR1 (5′-TAAACTTCTGGATGTCCAAAAAATCA-3′) [39]. The PCR cycle was performed in a C1000^TM^ Thermal Cycler (Bio-Rad, Hercules, CA, USA) with a total reaction volume of 50 μL containing: 5 μL of 10× PCR buffer, 2.50 μL of 50 mM MgCl_2_, 36.01 μL of distilled water, 0.25 μL of 10 mM dNTPs, 0.24 μL of 1.2 unit of platinum *Taq* polymerase (Invitrogen, Carlsbad, CA, USA), 0.50 μL of 10 mM forward primer, 0.50 μL of 10 mM reverse primer, and 5 μL of DNA template. The PCR cycling conditions consisted of initial denaturation at 94 °C for 1 min; 5 cycles of 94 °C for 30 s, annealing at 45 °C for 40 s, and extension at 72 °C for 1 min; 30–35 cycles of 94 °C for 30 s, 55 °C for 40 s, and 72 °C for 1 min; final extension at 72 °C for 10 min; followed by indefinite hold at 4 °C. Amplified PCR products were separated by gel electrophoresis on 2% agarose gel, with a GeneRuler™ 100 bp DNA ladder (Thermo Scientific, Vilnius, Lithuania), which was used to estimate the size of DNA fragments on the gel. The voucher specimens are deposited in the collection of the Faculty of Veterinary Science, Mahidol University (VSMU), Thailand.

#### 2.3.2. DNA Sequencing and Data Analysis

DNA sequencing of PCR products was performed using an ABI 3730 XL DNA analyzer at Bioneer (Daejeon, Korea) with LepF1 and LepR1 primers. The COI sequences were edited and analysed using MEGA 6.0 (Molecular Evolutionary Genetics Analysis) software [40]. Multiple alignments of all nucleotide sequences were done using ClustalW, available within MEGA 6.0. All sequences obtained in this study were 100% identical. A single representative sequence was deposited in GenBank under the accession number MN883828. To confirm the species identification, the representative sequence was blasted against the GenBank database using the nucleotide BLAST (available at https://blast.ncbi.nlm.nih.gov/Blast.cgi) and compared with the Barcode of Life Data Systems (BOLD) using the BOLD Identification System (IDS) (available at http://v4.boldsystems.org/index.php/IDS_OpenIdEngine). A phylogenetic tree based on neighbour joining (NJ) was constructed with the Kimura two-parameter (K2P) model and bootstrapping (1000 replicates) in MEGA 6.0. The sequences of stomoxyine flies available in GenBank were included in the analysis (Table 1)*. Musca domestica* Linnaeus, 1758 (Diptera: Muscidae) KM571920 was selected as an outgroup.

### 2.4. Geometric Morphometric Analysis

#### 2.4.1. Wing Preparation

The left wing of males and females of *H. aberrans* was dissected from the body and mounted with Hoyer’s medium on microscope slides. All slides were photographed using a digital camera connected to a stereomicroscope (Nikon AZ 100, Nikon Corp, Tokyo, Japan). A total of 16 wing pictures (9 males and 7 females) were analysed using the landmark-based method.

#### 2.4.2. Landmark-Based Method

**Figure 2:**
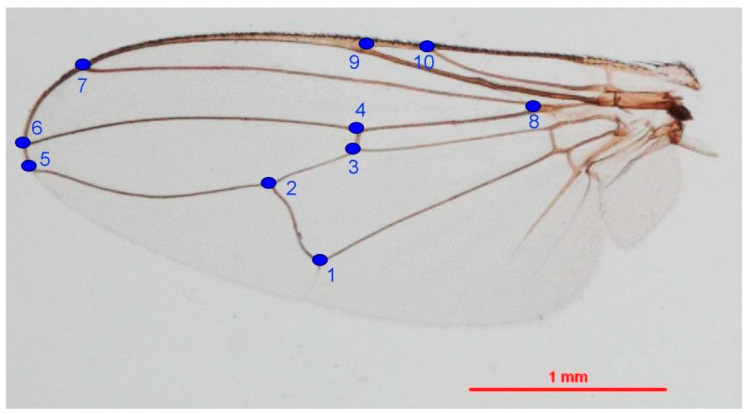
Ten landmarks digitised on wings of *Haematobosca aberrans* Pont, Duvallet & Changbunjong, 2020.

The coordinates of ten wing landmarks (Figure 2) based on the study of Changbunjong et al. (2016) [26] were digitised for geometric morphometric analysis. The wing size was estimated using the centroid size (CS) derived from coordinates of all landmarks. The centroid size is defined as the square root of the sum of the squared distances between the centre of the configuration of landmarks and each separate landmark [47]; it was computed after Generalised Procrustes Analysis (GPA), also known as Procrustes superimposition [48]. The centroid size difference was compared between sexes by non-parametric tests based on 1000 permutations, with Bonferroni correction for a test of significance at *p*-value < 0.05. The wing shape variables were computed as principal components of the partial warp (PW) scores or relative warps (RWs); they were also computed after Generalised Procrustes Analysis. The Procrustes superimposition provided configurations for visual comparison of the mean anatomical landmarks between the sexes. The relative warps were used to explore the morphospace and used as an input for the discriminant analysis (DA) or canonical variate analysis (CVA), which was illustrated by the factor map. Mahalanobis distance between the sexes was calculated from DA analysis. The statistical significance of shape differences between sexes was obtained by non-parametric analyses based on 1000 permutations, with Bonferroni correction for a test of significance at *p*-value < 0.05. A cross-validated classification was used to test the accuracy of classification between sexes based on Mahalanobis distances. The allometric effect, the effect of size on shape variation, was performed by linear regression of the Procrustes shape coordinates on centroid size and then estimated by the determination coefficient and statistical significance of the model at *p*-value < 0.05.

#### 2.4.3. Morphometric Software

Geometric and multivariate analyses were performed using the CLIC package version 99 [25], freely available at https://xyom-clic.eu.

## 3. Results

**Figure 3:**
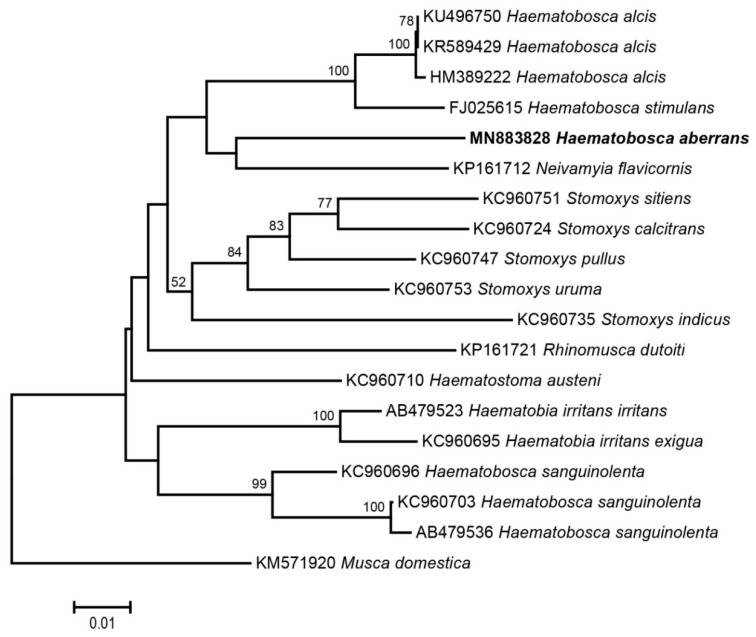
Neighbour-joining (NJ) tree based on Kimura two-parameter (K2P) model for cytochrome c oxidase I (COI) sequence of *Haematobosca aberrans* Pont, Duvallet & Changbunjong, 2020 collected from northern Thailand and sequences obtained from GenBank. Only bootstrap values ≥50% are shown. The scale bar represents 0.01% divergence.

A total of 16 specimens of *H. aberrans*, comprising 9 males (56.25%) and 7 females (43.75%), were collected in this study. For genetic analysis, only 10 specimens (5 males, 5 females) were used in the current study. A standardised 658 bp fragment of the mitochondrial COI gene was successfully amplified from all specimens. No stop codons, insertions or deletions were observed in these sequences. All sequences were 100% identical. The sequence had high A + T content (69.3%), with nucleotide composition of A = 30.2%, T = 39.1%, C = 14.4% and G = 16.3%. The sequence showed 92.52% similarity to *Neivamyia flavicornis* (Malloch, 1928) in GenBank but did not match any identified species in BOLD. The sequence of *H. aberrans* (MN883828) was used in the phylogenetic analysis. The phylogenetic relationship of the COI sequence of *H. aberrans* and other related species was established using an NJ tree (Figure 3). *Haematobosca aberrans* was clearly separated from other species within the subfamily Stomoxyinae.

**Figure 4:**
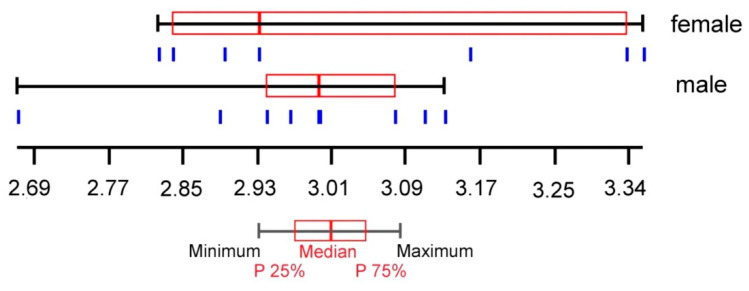
Centroid size variation of the wings between sexes of *Haematobosca aberrans* Pont, Duvallet & Changbunjong, 2020, shown as quartile boxes. Each box shows the group median separating the 25th and 75th quartiles. Vertical bars under the boxes represent the wing (units as mm).

**Figure 5:**
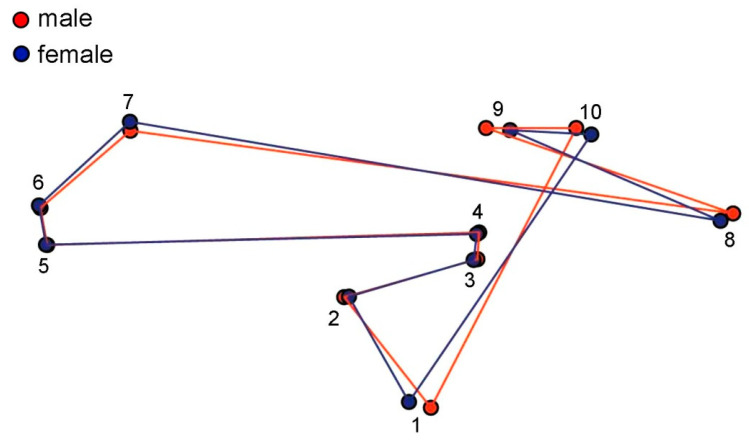
Configuration of the mean anatomical landmark positions connected by a straight line after Procrustes superimposition of *Haematobosca aberrans* Pont, Duvallet & Changbunjong, 2020 in both sexes.

**Figure 6:**
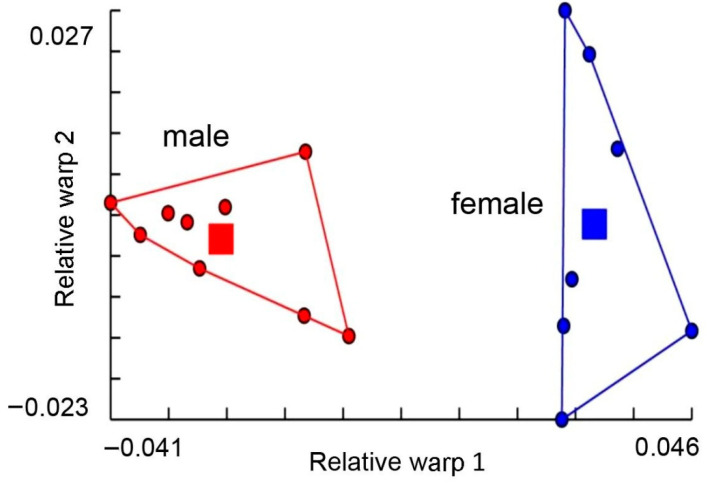
Morphospace of males and females of *Haematobosca aberrans* Pont, Duvallet & Changbunjong, 2020 based on wing shape variables. The horizontal axis is the first relative warp (RW1), and the vertical axis is the second relative warp (RW2).

**Figure 7:**
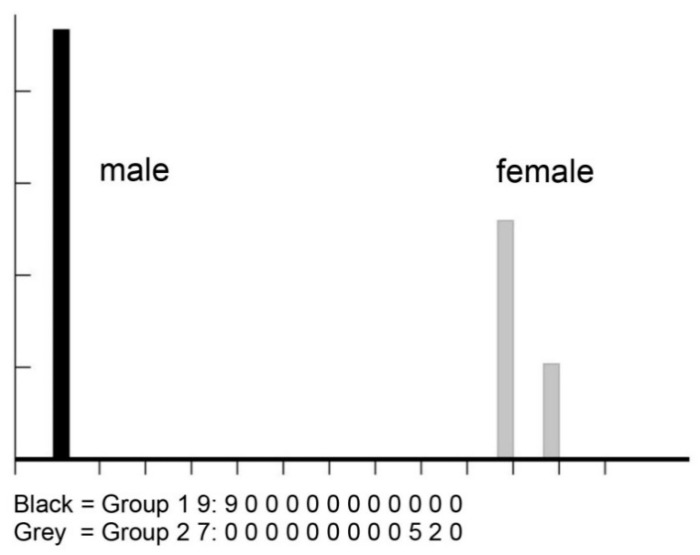
Factor map of the discriminant factor separating males (black bar) and females (grey bar) of *Haematobosca aberrans* Pont, Duvallet & Changbunjong, 2020.

For geometric morphometric analysis, a total of 16 wing pictures (9 males and 7 females) were analysed using the landmark-based approach. The wing size (centroid size) variation between sexes of *H. aberrans* is illustrated in Figure 4. The mean wing CS of males and females was 2.98 ± 0.13 mm and 3.05 ± 0.22 mm, respectively. There was no statistically significant difference between the sexes. The visual comparison of the mean anatomical landmark positions between the sexes showed most visible landmark displacements in the upper and lower parts of the wing (landmarks 1, 7, 8, 9, 10) (Figure 5). Morphospace based on wing shape variables from principal components and the factor map of the discriminant factors showed a clear difference between the sexes (Figure 6 and Figure 7). Based on the Mahalanobis distance, the wing shape was significantly different between the sexes of *H. aberrans* (Mahalanobis distance = 7.17). The accuracy score after a cross-validated classification test was 100% in both males and females. The wing shape has no allometric effect. The determination coefficient was 10% but was not statistically significant.

## 4. Discussion

Our study has revealed updated data about stomoxyine flies in Thailand. The new species *Haematobosca aberrans* was described recently in terms of its morphological characteristics [6] and was found in the Chiang Mai Province of northern Thailand. However, this species was apparently previously recorded in the Kanchanaburi Province of western Thailand but under the name *Stygeromyia* sp. [7]. Buffaloes may be important hosts of this fly in the studied area. However, buffaloes have also been reported to be hosts that can be attacked by the species *H. stimulans* [1], while other hosts of *Haematobosca* spp. include cattle, horses, wildlife and humans [1,2,3,5,49]. In the present study, the habitat of the buffalo farm consisted of a human settlement and of forest, which may provide suitable breeding sites for these flies. In the genus *Haematobosca*, only *H. sanguinolenta* had been reported in Thailand. This species was found in livestock farms, wildlife conservation areas and national parks [5]. In our present study, we found that *H. sanguinolenta* and *H. aberrans* could be found together in the same habitat of the livestock farm. These results suggest that livestock farms are sites with the greatest diversity of stomoxyine species [5].

The results obtained from this study showed that the alignment of COI sequences of *H. aberrans* was straightforward and these sequences lacking stop codons, insertions or deletions indicated that all sequences were functional mitochondrial COI sequences. The nucleotide sequences showed a high A + T content (69.3%), which is consistent with previous studies on stomoxyine flies (69%) [24]. One hundred percent similarity could be detected between the different *H. aberrans* sequences in the present study. This may be due to the low number of samples and/or to the fact that all the flies were collected from the one population. Comparison between our sequence and reference sequences in GenBank and BOLD databases can be used to confirm a new species of *H. aberrans* as previously described by Pont et al. (2020) [4]. The phylogenetics based on the NJ tree indicated that the COI gene could distinguish among stomoxyine species.

There is a correlation between the morphological characters already described [6] and the molecular results presented in our study. *H. aberrans* could be separated from other species by morphological characters. However, *H. aberrans* and *N. flavicornis* were clustered together in the phylogenetic tree but they received low bootstrap support. Based on the morphological characters, *H. aberrans* and *N. flavicornis* are easily distinguished by their strap-like palpi, which are not grooved internally in *N. flavicornis* but are more dilated terminally and grooved internally in *H. aberrans*. In addition, the anterior katepisternal seta is absent in *H. aberrans* but present in *N. flavicornis* [1,3]. Within the genus *Haematobosca*, the main morphological character distinguishing *H. aberrans* from other species is the absence of the anterior katepisternal seta [6]. According to the key by Zumpt (1973) [1], the species *H. aberrans* resembles species of the genus *Stygeromyia* Austen, 1907 because the anterior katepisternal seta is absent. However, *H. aberrans* has long hairs on the upper and lower sides of the arista, as in other species of *Haematobosca*, and this differs from *Stygeromyia* where there are long hairs only on the upper side. In addition, the meron and prosternum are bare in *H. aberrans* and other *Haematobosca* but are setulose in *Stygeromyia* [6]. So far as the genetics are concerned, there is little information on *Haematobosca* and other stomoxyine species, especially *Stygeromyia* spp., in the GenBank database that is available for comparison with our new sequences.

The geometric morphometric study was carried out to compare the wing size and wing shape between sexes of *H. aberrans*. Landmark-based geometric morphometrics has been mostly used in sexual dimorphism studies [32,33,36]. In the present study, we revealed a sexual dimorphism in wing shape, indicating that the phenotypic expression of wing shape is a sex-specific difference. This is consistent with a previous report on stomoxyine flies: *Stomoxys indicus*, *S. pullus* and *S. uruma* [26]. The sexual dimorphism of wing shape has been reported in other insect vectors such as mosquitoes of the genus *Aedes* (*Ae. aegypti* (Linnaeus, 1762), *Ae. albopictus* (Skuse, 1894)), *Anopheles* (*An. albitarsis* Lynch Arribalzaga, 1878, *An. cruzii* Dyar & Knab, 1908, *An. homunculus* Komp, 1937, *An. strodei* Root, 1926, *An. triannulatus* Komp, 1937), *Culex* (*Cx. quinquefasciatus* Say, 1823, *Cx. nigripalpus* Theobald, 1901) and *Ochlerotatus* (*Oc. scapularis* (Rondani, 1848)) [34]. Furthermore, sexual dimorphism of both wing size and wing shape has also been found in some mosquito species: *Mansonia bonneae* Edwards, 1930 and *M. dives* (Schiner, 1868) [33]. The sexual size dimorphism may be mainly affected by environmental factors such as food quantity/quality or temperature [50], while sexual shape dimorphism is often a passive consequence of sexual size dimorphism (allometry) [33]. The sexual shape dimorphism in our study is not correlated with size (non-allometric effect). However, non-allometric effects were probably playing a role in the sexual shape dimorphism of *H. aberrans*. According to Gidaszewski et al. (2009) [37], flight behaviour and mating systems may be related to variations in wing shape.

## 5. Conclusions

This study has provided genetic and phenotypic information on *H. aberrans* in Thailand. The COI gene can be used to differentiate *Haematobosca* spp. and other related species within the subfamily Stomoxyinae. It has also confirmed that *H. aberrans* is distinct and different from the other known species. Landmark-based geometric morphometrics is an effective way of separating between the sexes of this species. The sexual shape and size dimorphism could be a basis for further studies on the influence of sex-specific divergence in response to environmental conditions and the species identification of *Haematobosca* spp. Due to the small number of specimens in this study, further studies including more specimens are recommended to increase the reliability of wing shape for sexual discrimination. Also, behavioral and ecological studies are required to confirm the existence of sexual dimorphism. Additionally, a more comprehensive phylogeny with various gene markers, different populations of the same species, further species from each genus, and other related species will provide additional information on genetic relationships within the Stomoxyinae.

## Figures and Tables

**Table 1:** Stomoxyine flies from the GenBank database used for constructing the phylogenetic tree.

Species	Origin	Accession Number	Reference
*Haematobia irritans irritans* (Linnaeus, 1758)	Japan	AB479523	[41]
*Haematobia irritans exigua* de Meijere, 1906	Thailand	KC960695	[24]
*Haematobosca alcis* (Snow, 1891)	Canada	HM389222, KR589429	[42,43]
	USA	KU496750	[44]
*Haematobosca sanguinolenta* (Austen, 1909)	Thailand	KC960696, KC960703	[24]
	Vietnam	AB479536	[41]
*Haematobosca stimulans* (Meigen, 1824)	NA	FJ025615	[45]
*Haematostoma austeni* Malloch, 1932	Thailand	KC960710	[24]
*Neivamyia flavicornis* (Malloch, 1928)	Brazil	KP161712	[46]
*Rhinomusca dutoiti* Zumpt, 1950	South Africa	KP161721	[46]
*Stomoxys calcitrans* (Linnaeus, 1758)	Thailand	KC960724	[24]
*Stomoxys indicus* Picard, 1908	Thailand	KC960735	[24]
*Stomoxys pullus* Austen, 1909	Thailand	KC960747	[24]
*Stomoxys sitiens* Rondani, 1873	Thailand	KC960751	[24]
*Stomoxys uruma* Shinonaga & Kano, 1966	Thailand	KC960753	[24]

NA, no available data.
